# Metal-organic framework impregnated sponge-based TENG as a binary input device for logic gate simulation and power plant control

**DOI:** 10.1186/s11671-025-04369-6

**Published:** 2025-10-30

**Authors:** Nitha Palakkattiri Krishnan, Gaurav Khandelwal, Arunkumar Chandrasekhar

**Affiliations:** 1 Nanosensors and Nanoenergy Lab, Biomedical instrumentation Lab, Department of Sensors and Biomedical Technology, School of Electronics Engineering, Vellore Institute of Technology, Vellore, Tamilnadu India; 2 Engineering and Technology Institute (ENTEG), University of Groningen, Groningen, Netherlands

**Keywords:** MIL-53, TENG, Energy harvesting, Logic gates, Power plant control

## Abstract

Logic gates are the essential components of digital electronics. These are the electronic circuits that generate a single binary output by applying fundamental logical operations on inputs. These are the basic building blocks of both combinational and sequential circuits. Different approaches are available to implement logic operations, among which a novel method utilizes a Triboelectric nanogenerator (TENG), which converts mechanical energy into electrical energy by using Maxwell’s equations and can act as a trigger device corresponding to mechanical stimuli. TENGs can act as a binary input device for logic gate operations, implementing logic 0 corresponding to ‘NO PRESS’ or out of contact and logic 1 corresponding to the TENGs ‘PRESS’ or in contact state. Herein, a metal organic framework (MOF) MIL-53 impregnated sponge is used for the TENG device fabrication. MIL-53 is an important member of the MOFs, characterized by a large surface area (25–270 m^2^/g), high adsorption rate, and tunable pore size with a positive surface charge. The TENG device is constituted using MIL-53(MS-TENG) and analyzed in contact separation mode. The highest performance is achieved with the 4 wt.% filling of MIL-53 particles. The MS-TENG generated an output voltage of 44.2 V, current of 0.9 µA, and the transferred charge of 6 nC. The device is used for simulating various logic gates and implementing a half-adder using LabVIEW software with MS-TENG as the input device. Since ladder logic is the basic principle of power plant operations, MS-TENG is used to trigger (simulation using LabVIEW) various power plant control operations. This approach unveils the industrial application using MOF impregnated TENG.

**Supplementary Information:**

The online version contains supplementary material available at 10.1186/s11671-025-04369-6.

## Introduction

Metal-organic frameworks are porous crystalline materials characterized by a high surface area and tunable pore sizes. In MOFs, a metal ion is coordinated to an organic ligand, where the former is selected from transition metals and the latter is from organic linkers like benzene-1,4-dicarboxylic acid (terephthalic acid, BDC), biphenyl-4,4’-dicarboxylic acid (BPDC), isophthalic acid, and trimesic acid (benzene-1,3,5-tricarboxylic acid). The MOFs has wide range of applications including gas storage, gas separation, energy harvesting, and water harvesting etc. MOFs are extensively used for energy harvesting via triboelectric effect [1–3]. TENG converts mechanical energy into electrical energy, utilizing a wide range of materials by coupling of contact or triboelectrification and electrostatic induction [4–7].

Matériaux de l′Institut Lavoisier (MIL) is a MOF subclass widely used for gas storage and adsorption. MIL 88-A is one of the family members that has already been used for the TENG formation [8]. The notable properties of this material are biodegradability and non-toxicity. The electrical performance of the MIL-TENG device, in which MIL-88 A was used as a positive active material with FEP as a negative material is summarised inTable.1. Similarly amine functionalized MIL-101 TENG is used with different wt% of the cellulose nanofiber, thus improving the electrical performance by 230% compared with the one without functionalization [9]. The charge induction and trapping properties of the amine group are deployed here.

MIL-53 is another important member of MIL series, featuring M-OH chains and a terephthalate linker connected through a coordination bond [10], where M represents the Metal (Al, Fe, or Cr) in MIL-53. Six oxygen atoms coordinate octahedrally around each metal center [11, 12]. The remaining two oxygen atoms are part of two distinct nearby metal centers, while the other four oxygen atoms come from four distinct carboxylate groups. One-dimensional diamond-shaped pores are found in the framework structure [13]. Herein, MIL-53(Fe) is introduced for TENG applications using an ecoflex sponge. MIL-53 acts as a filler with a positive zeta potential of + 7.7 mV, which enhances the performance of the ecoflex sponge due to the charge-trapping property of MOFs. The sponge is prepared by the sugar sacrificial template method [14], and MIL-53 microparticles are incorporated inside the pores of the sponge. MS-TENG is fabricated in a contact separation mode, with MIL-53 sponge as the negative layer and aluminium as the positive layer, respectively. There are dual tribo layer formations here that are effective; one between Al and the sponge, and the other between MIL-53 and the walls of the sponge. MS-TENG achieved a maximum voltage of 44.2 V, a short-circuit current of 0.9 µA, and a transferred charge of 6 nC. The instantaneous power and power density are 7.6 µW and 1.9 µW/cm^2^, respectively. MS-TENG is used for charging various capacitors and is utilized in energy harvesting applications, such as driving low-power electronic devices like LEDs and LCDs.

The MS-TENG is also demonstrated for logic operations implementation as an input device, with the integration of LabVIEW software (simulation). Basic operations, such as NOT, AND, NAND, OR, NOR, and XOR, are implemented using simulation. Further, a half-adder implementation is also performed with MS-TENG. Since the fundamental principle of Programmable Logic Controller (PLC) and Supervisory Control and Data Acquisition(SCADA)-controlled power plant controls is ladder logic [15], it can be implemented here using TENG as an input device. Power plant coolant operation and conveyor belt operations are also executed using LabVIEW software. Thus, the MS-TENG is a potential candidate for implementing various power plant control operations and industrial applications by integrating LabVIEW software.

## Materials and methods

### Materials

Ferric chloride hexahydrate (FeCl_3_.6H_2_O), terephthalic acid (H_2_-BDC), and dimethylformamide (DMF) were purchased from Sigma-Aldrich. Chemicals were used directly without any treatment. Ecoflex 00–30 platinum silicone rubber compound (1:1) is used to prepare the sponge.

### Synthesis procedure of MIL-53

**Fig. 1:**
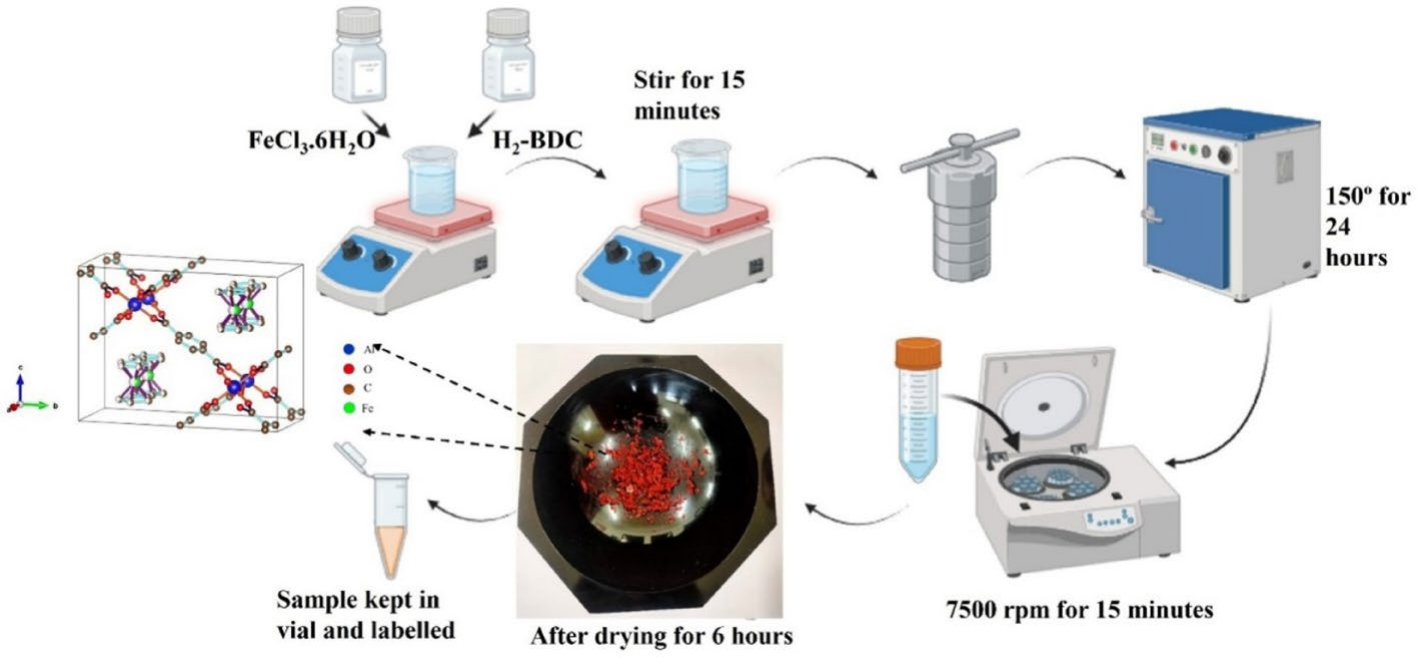
Schematic illustration of material synthesis procedure

As illustrated in Fig. 1, MIL-53 is synthesized using an previously documented solvothermal process [16, 17]. Here, the reaction’s temperature and duration are optimized. In DMF, 0.68 g (2.5 mmol) of ferric chloride (FeCl_3_.6H_2_O) and 0.42 g (2.5 mmol) of terephthalic acid (H_2_-BDC) were dissolved by stirring. The solution was then transferred to a 100 mL Teflon container and subsequently placed in a stainless-steel autoclave. The autoclave was placed in an oven at 150 °C for 24 h. (Reaction was conducted under various conditions as specified in Table S1, and the desired product was formed under condition three). The autoclave was allowed to cool. The brown-colored solid powder was collected by centrifugation and rinsed with DMF and ethanol. Subsequently, it was dried at 60 °C for 24 h. The powder was grounded as illustrated in Fig. 1. The simulated structure of MIL-53, is shown in Fig. 1.

### Material characterization

Powder X-ray Diffraction (PXRD), Fourier Transform IR (FT-IR) spectroscopy, Field Emission Scanning Electron Microscopy (FE-SEM), and Zeta potential analysis were employed to confirm the crystalline nature, morphology, and zeta potential of MIL-53. The iron-filtered Cu Kα radiation (λ = 1.5406 Å) is employed to record PXRD in the 2θ range of 5°- 35° with a step size of 0.02° and a time of 0.3 s per step using Bruker D.S. advance apparatus. Thermo Fisher Nicolet iS50 is employed to perform FT-IR. FE-SEM was carried out with a Thermo Fisher FEI QUANTA 250 FEG, which is capable of operating within the 5 kV–30 kV voltage range and achieves a high resolution of 1.2 nm at 30 kV in high vacuum conditions. The Anton Paar Litesizer 500 instrument is employed to conduct zeta potential analysis. The H5KS model of a universal testing equipment from Tinius Olsen, UK, is used to conduct stress-strain analysis.

### Fabrication of pristine sponge TENG (PS-TENG) and MIL- 53 sponge TENG (MS-TENG)

**Fig. 2:**
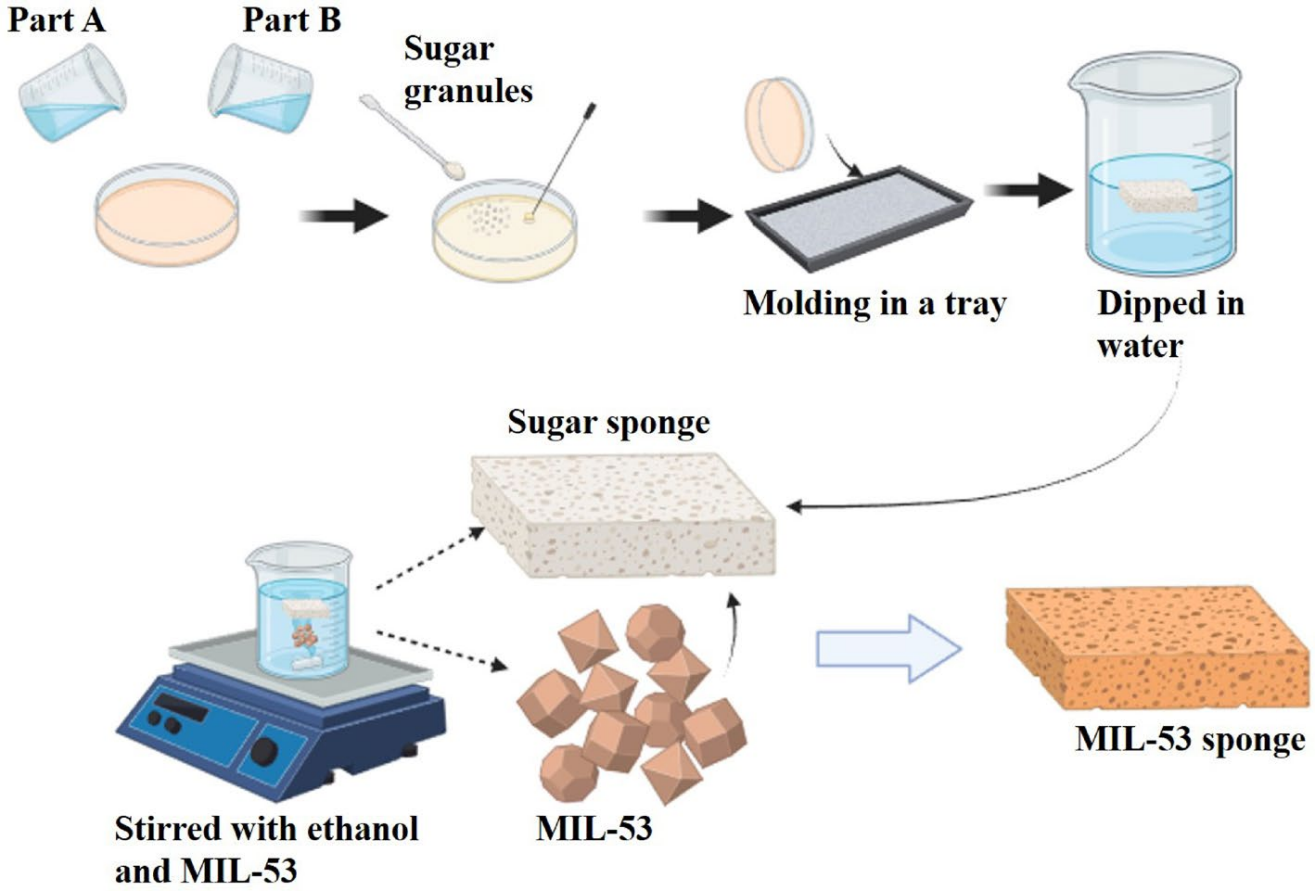
Preparation of sponge via Sacrificial template method

A sugar sacrificial template method was employed to create a pristine ecoflex sponge [14, 18]. Parts A and B of the Ecoflex 00–30 platinum silicone rubber compound are mixed in a 1:1 ratio for this purpose. The mixture is continuously stirred, and micro cuboidal sugar granules are subsequently introduced and agitated for an additional two minutes, as illustrated in Fig. 2. The solution was poured into a molding tray and allowed to cure overnight. The sugar crystals were removed by immersing the ecoflex layer in water for two hours after which it was peeled off. MIL-53 sponge was prepared by placing ecoflex sponge in a MIL-53 dispersion for 12 h. Subsequently, the ethanol evaporated, and the MIL-53 powder was incorporated into the pores of the ecoflex sponge to create the MIL-53 sponge. In contact separation mode TENG (PS-TENG and MS-TENG) are formed by utilizing Pristine sponge and MIL-53 sponge. The sponge serves as the negative material, while aluminum serves as the positive material. The pristine sponge and MIL-53 sponge have dimensions of 2 cm × 2 cm × 0.5 cm.

### Electrical performance evaluation

Keithley electrometer 6514 is used to measure peak to peak voltage (Vp-p), short circuit current (I_SC_) and the transferred charge(Q) of both PS-TENG and MS-TENG.

## Results and discussion

**Fig. 3:**
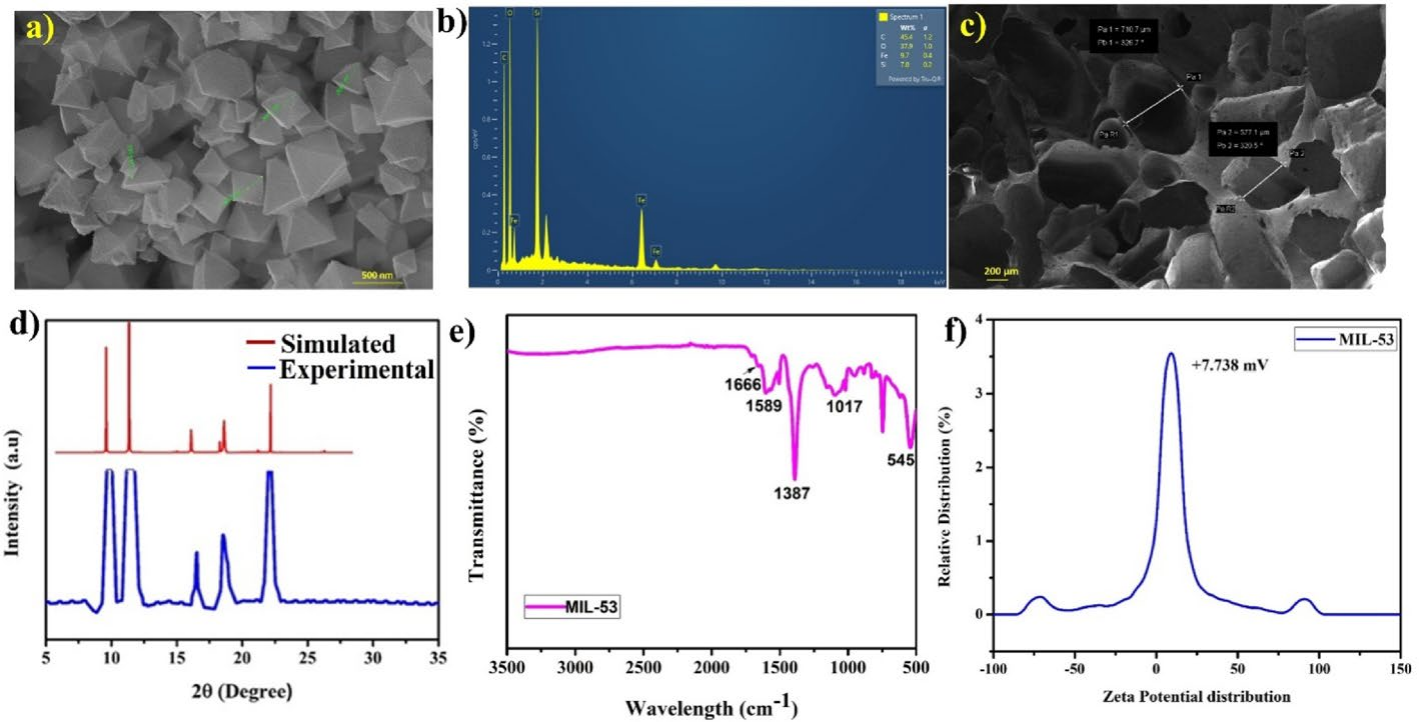
Physiochemical characterization of MIL-53 (**a**) FE-SEM image. (**b**) Energy Dispersive Spectroscopy. (**c**) SEM image of pristine sponge structure. (**d**) PXRD pattern of MIL-53. (**e**) FTIR spectra of MIL-53. (**f**) Zeta potential of MIL-53

The precursors for MIL-53 are ferric chloride hexahydrate (FeCl_3_.6H_2_O) and terephthalic acid. Temperature and reaction time are the two parameters that are optimized to ensure the formation of MOFs. Table S1 lists the three distinct conditions under which reactions were conducted. The third condition resulted in the production of MIL-53, as verified by XRD and FTIR. Figure 3.a illustrates the FE-SEM image of MIL-53, which demonstrates an octahedral morphology with a high degree of uniform particles. Fig. S1 illustrates the FE-SEM image of MIL-53, synthesised at 150 °C for 15 h. The image confirms the presence of numerous unreacted particles. Consequently, the reaction period was extended to 24 hours, and MIL-53 was successfully synthesized without any unreacted particles as confirmed by FE-SEM. The presence of carbon, oxygen, and iron in the MIL-53 is confirmed by the energy dispersive spectroscopy (EDS) results, as illustrated in Fig. 3b. Figure 3c shows the SEM image of the pristine Ecoflex sponge. The Ecoflex sponge has a pore size of 500–700 μm.

PXRD analysis is performed to examine the crystalline structure by analyzing diffraction peaks. The XRD pattern of MIL-53 is depicted in Fig. 3.d, which spans the 2θ region from 5º to 35º. The diffraction peaks are present at 9.9º (011), 11.43º (020), 16.5º (101), 18.55º (121), and 22.06º (013), and this is in good agreement with previously reported results [19–21]. The XRD simulated pattern is shown in red, and it align with the experimental XRD pattern in blue, as shown in Fig. 3.d. Therefore, it can be inferred that the MIL-53 that was synthesized is highly crystalline. FT-IR spectroscopy was employed to detect the molecular structure and functional group bonding between the constituents in MIL-53. Figure 3e depicts the FT-IR spectra of MIL-53 in the range 500 to 3500 cm^− 1^. The spectra indicate characteristic peaks in the 1400–1700 cm^− 1^ range, which correspond to the carbonyl (CO) group (1666 cm^− 1^) and the stretching vibration of the carboxyl (COOH) groups with the Fe metal nodes (1589 cm^− 1^). The benzene ring in the H_2_-BDC exhibits C-H bending vibrations at 747 cm^− 1^. The peak at 545 cm^− 1^ corresponds to the Fe-O bond, indicating the formation of the M-OH bond between the metal Fe and the organic ligand terephthalic acid [22]. Figure 3f shows the zeta potential distribution of the MIL-53 dispersion. The zeta potential is found to be + 7.7. mV as depicted in the graph and surface potential should be higher than that. The positive zeta potential value suggests that MIL-53 can function as a positive triboelectric material.

The PS-TENG and MS-TENG devices were fabricated using the process detailed in Sect. 2.4. The photograph of the sponge is shown in Fig. S2. A microscopic image was acquired to verify that MIL-53 is incorporated within the pores of the sponge. In Fig. S3 shows the microscopic images of pristine and MIL-53 impregnated sponges. The microscopic images confirmed that MOF particles are attached to the inner pores of the sponge, which may enhance the performance of the sponge. Further, the microscopic image of the MIL-53 sponges at various magnifications (10x, 20x, and 40x) are depicted in Fig. S4. MIL-53 is brown-coloured particles, distributed inside the pores so that a TENG formation occurs inside the sponge itself, where MIL-53 is positive and the walls of the pore are negative layers of the TENG. The SEM image of the MOF-incorporated sponge is shown in Fig. S5, which reveals that the MOF filler is attached to the walls of the sponge, making the edges of the sponge invisible.

**Fig. 4:**
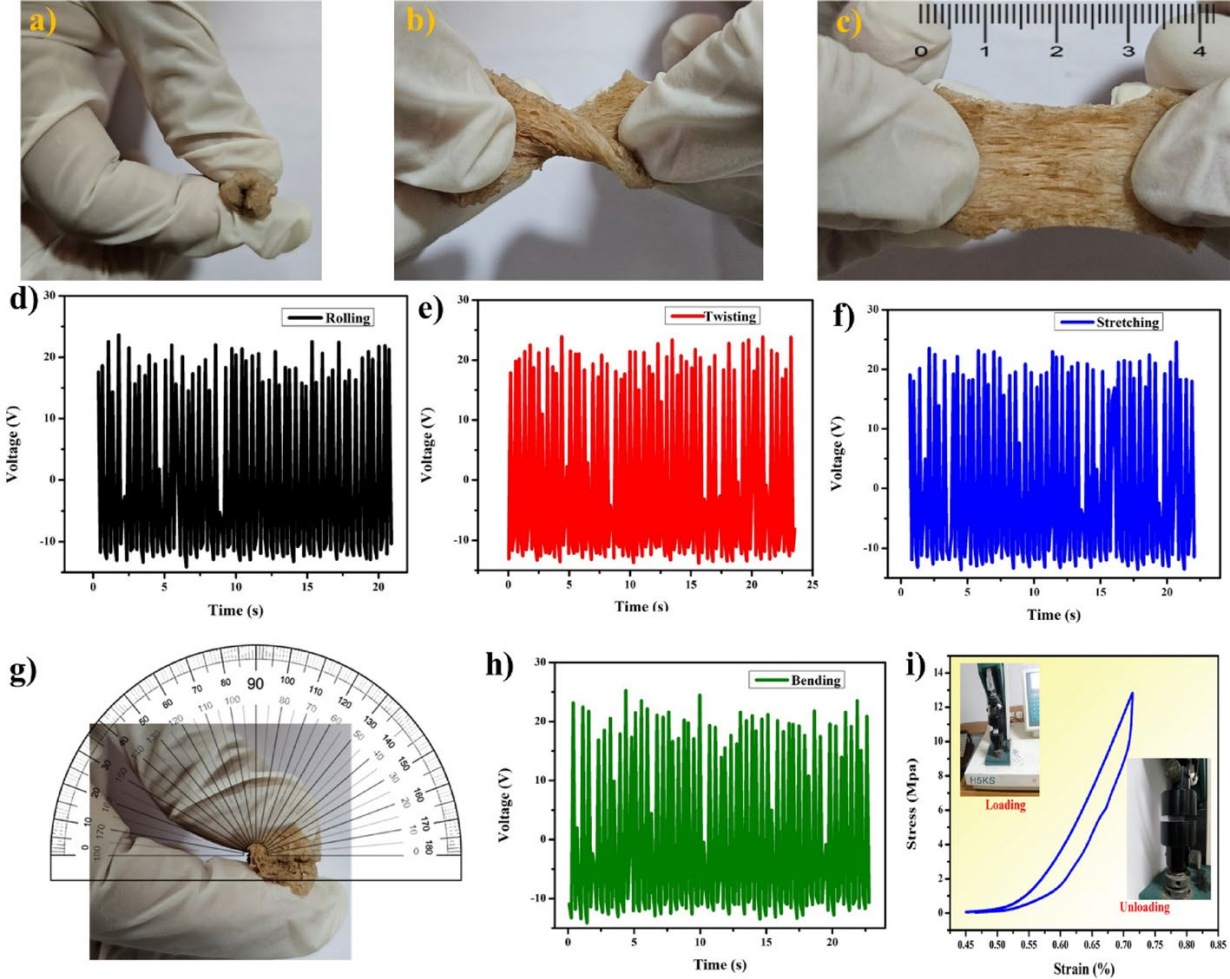
Mechanical properties and electrical response of MIL-53 sponge. (**a**) Rolling. (**b**) Twisting. (**c**) Stretching. (**d**–**f**) Electrical performance after performing mechanical actions. (**g**) Bending. (**h**) Electrical performance after bending. (**i**) Stress-strain plot

The MIL-53 sponge is highly flexible under rolling, twisting, stretching, and bending (Fig. 4 (a-c, g)). In Fig. 4 (c), the sponge is stretched to a maximum of 3 cm and in Fig. 4 (g) the bending angle is 180°. After the film is subjected to mechanical activities, such as bending, it is utilized to construct the device in contact separation mode, and the output voltage is measured. The efficacy is consistent throughout, as illustrated in Fig. 4 (d-f, h). Consequently, MIL-53 sponge is appropriate for electronic applications that are both flexible and wearable. Figure 4i illustrates the stress–strain curve that was generated subsequent to the parameter configuration in the software. The universal material testing machine (Fig. S6) was employed to conduct a compressibility test on the sponge. The test conditions consisted of an initial specimen height of 5 mm, a displacement of 10 mm, an approach speed of 1 mm/min, and a maximal load capacity of 5145 N. The sponge exhibited a compressive strength of 13 MPa and exhibited outstanding loading–unloading behaviour without any visible damage [18, 23].

**Fig. 5:**
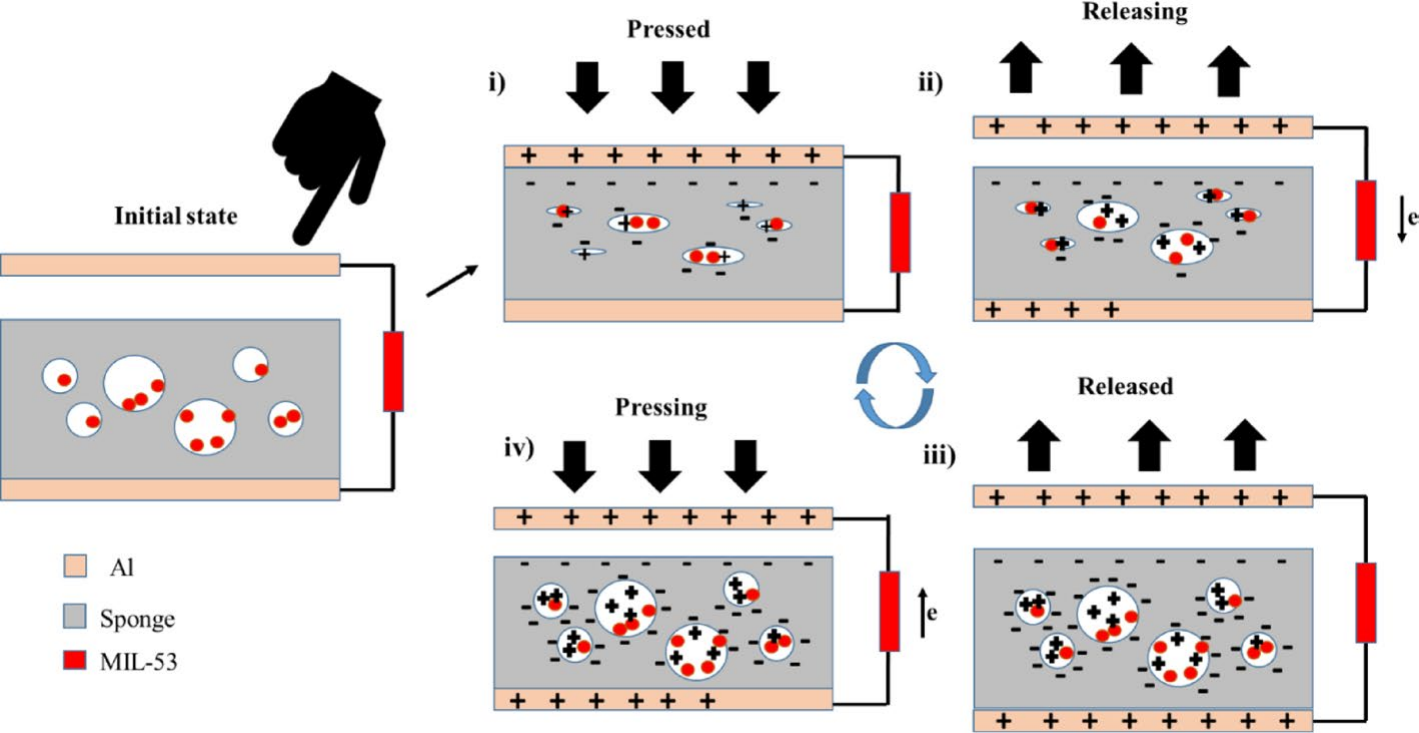
Working mechanism of MS-TENG

MS-TENG is designed in the contact separation mode, with sponge serves as the negative material and aluminum serves as the positive material. The sponge’s pores are effectively filled with MIL-53 microparticles, resulting in the formation of a triboelectric layer in the sponge i.e. between the MIL-53 particles and the sponge. Thus MS-TENG has two contacts one between the aluminium and the MIL-53 impregnated sponge, and second layer between the sponge wall and the impregnated MIL-53 particles. The pores of the sponge are compressed, contact electrification occurs, and opposite charges are generated when force is applied, as illustrated in Fig. 5 (i). When the force is released, pores gradually revert to their original state. There is a charge imbalance between the tribo layers, as the positive MIL-53 induces a negative charge in the sponge walls as a result of electrostatic induction. This imbalance leads to the electron flow from the top layer to the bottom layer, as illustrated in Fig. 5.ii. It will persist until the maximal separated state is achieved, at which point all pores are released, as illustrated in Fig. 5iii.

When force is applied again, the potential difference changes, causing electrons to move in the opposite direction, as shown in Fig. 5(iv). Positive and negative half cycles of AC will be generated by this continuous contact and separation. The performance enhancement of MIL 53-sponge (M-sponge) may be attributed to the presence of MIL-53, which induces interfacial polarization in the sponge, thereby forming a micro capacitor at the surface [24]. The MIL-53-impregnated sponge generates dual charge interfaces and as a result, the interfacial micro-capacitors act as localized voltage boosters, thereby enhancing the triboelectric yield beyond the capacity of pristine sponges. A synergistic tribo layer structure is established by the strategic impregnation of MIL-53, which leads to stable charge trapping and enhanced output performance that is suitable for industrial control systems and wearable applications [25].

Table 1 shows the benchmarking against prior work. From the table it is clear that, although MS-TENG has a lower peak output than MIL-88 A, it outperforms previous work in terms of multifunctionality, flexibility, and interaction with real-time control systems. Moreover, the proposed research consists of dual triboelectric layers, which has the advantages like higher surface charge density, and improved energy conversion efficiency. There are two interface layers here, one is between MIL-53 sponge and aluminium, and the other is between MIL-53 particles and the walls of the sponge as highlighted in the Table 1 (given in bold). Such a dual interface is the peculiarity of the state of the art of this research.

**Table 1:** Benchmarking against prior works

Positive material	Negative material	Mode of operation	Voltage (V)	Current (µA)	Power density (µW/cm^2^)	Application	No of contact interfaces	References
MIL-88 A	FEP, Kapton, and Ethyl cellulose	Contact separation	80	2.2	1.62	Powering of low-power electronics	Single	[8]
MIL-101	FEP	Contact separation	220	21	20.3	Energy harvesting	Single	[9]
ZIF-8 @wood	PDMS@wood	Contact separation	24.3	0.32	7.3	Smart building to power household devices	Single	[26]
ZIF-62	Teflon, Kapton, EC	Contact separation	62 V	1.4	0.968	Fitness tracking and powering of low-power electronics	Single	[12]
ZIF-67	Teflon/PDMS	Contact separation	118	0.17	15	Gait analysis and powering the robotic arm for identifying objects	Single	[7]
ZIF-7, ZIF-9, ZIF-11, and ZIF-12	Kapton/EC	Contact separation	60	1.1	4.84	Powering of low-power electronics	Single	[27]
**Aluminium**	**MIL53/Ecoflex sponge**	Contact separation	**44.2**	**0.9**	**7.6**	**Logic gate simulations and powerplant control**	**Dual**	**This work**

**Fig. 6:**
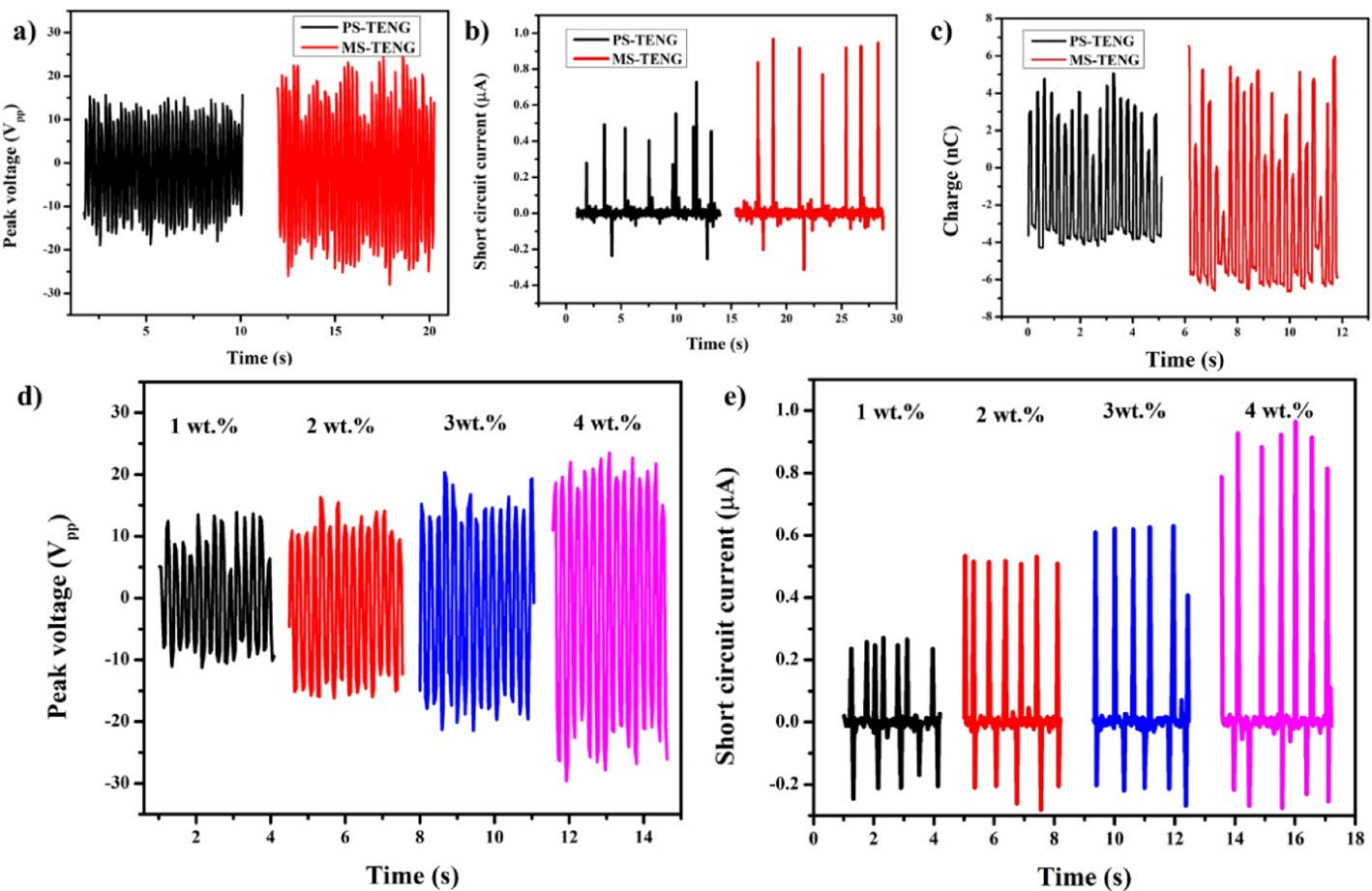
Electrical performance analysis of MS-TENG (**a**) Peak voltage (**b**) Short circuit current (**c**) Transferred charge of MS-TENG and PS-TENG. (**d**) Peak voltage and (**e**) Short circuit current of MS-TENG in different wt% of MOF impregnation

Figure 6 illustrates the performance analysis of the MS-TENG. The digitized photographs of the PS-TENG and MS-TENG are presented in Fig. S7. Performance analysis primarily focuses on three parameters: peak voltage, short-circuit current, and transferred charge. The highest peak-to-peak voltage (V_p−p_) for the MS-TENG is approximately 44.2 V, while the PS-TENG exhibits a value of around 30 V, as illustrated in Fig. 6.a. The irregular distribution of the filler results in voltage non-uniformity, potentially leading to localized charge leakage paths and a subsequent reduction in generated voltage. Likewise, irregular distribution of dielectric fillers might induce variations in capacitance, hence influencing the output of the TENG [28]. The maximum current obtained is 0.9 µA for MS-TENG and 0.4 µA for PS-TENG (Fig. 6.b), with the transferred charge being 6 nC for MS-TENG and 3 nC for PS-TENG (Fig. 6.c). In comparison to pristine material, M-sponge enhances performance owing to the charge-trapping characteristics of metal-organic frameworks (MOFs) [1, 28]. Furthermore, the MIL-53 filler enhances the electronegative characteristics of Ecoflex by internal charge transfer, as the internal surface area expands with the addition of the filler [29]. Various weight percentages of MOF microparticles contained into the sponge, as illustrated in Fig. S8. Voltage and current fluctuated based on the concentration of MIL-53 filler, with 4 wt.% of MIL-53 identified as the optimal loading for the sponge. The peak-to-peak voltage (V_p−p_) increased from 20 V to 44.2 V when the concentration of MIL-53 increased from 1 to 4 wt.%, as illustrated in Fig. 6.d. The 5 wt.% MIL-53-filled sponge exhibits a voltage of around 32 V, which is lower than the 4wt.% loading. A similar tendency is likewise noted in short-circuit current. The short-circuit current are 0.2 µA, 0.5 µA, 0.62 µA, and 0.9 µA, which correspond to 1 wt.%, 2 wt.%, 3 wt.%, and 4 wt.% loadings, respectively, as illustrated in Fig. 6 .e. The current decreases with additional elevations in filler concentration.

**Fig. 7:**
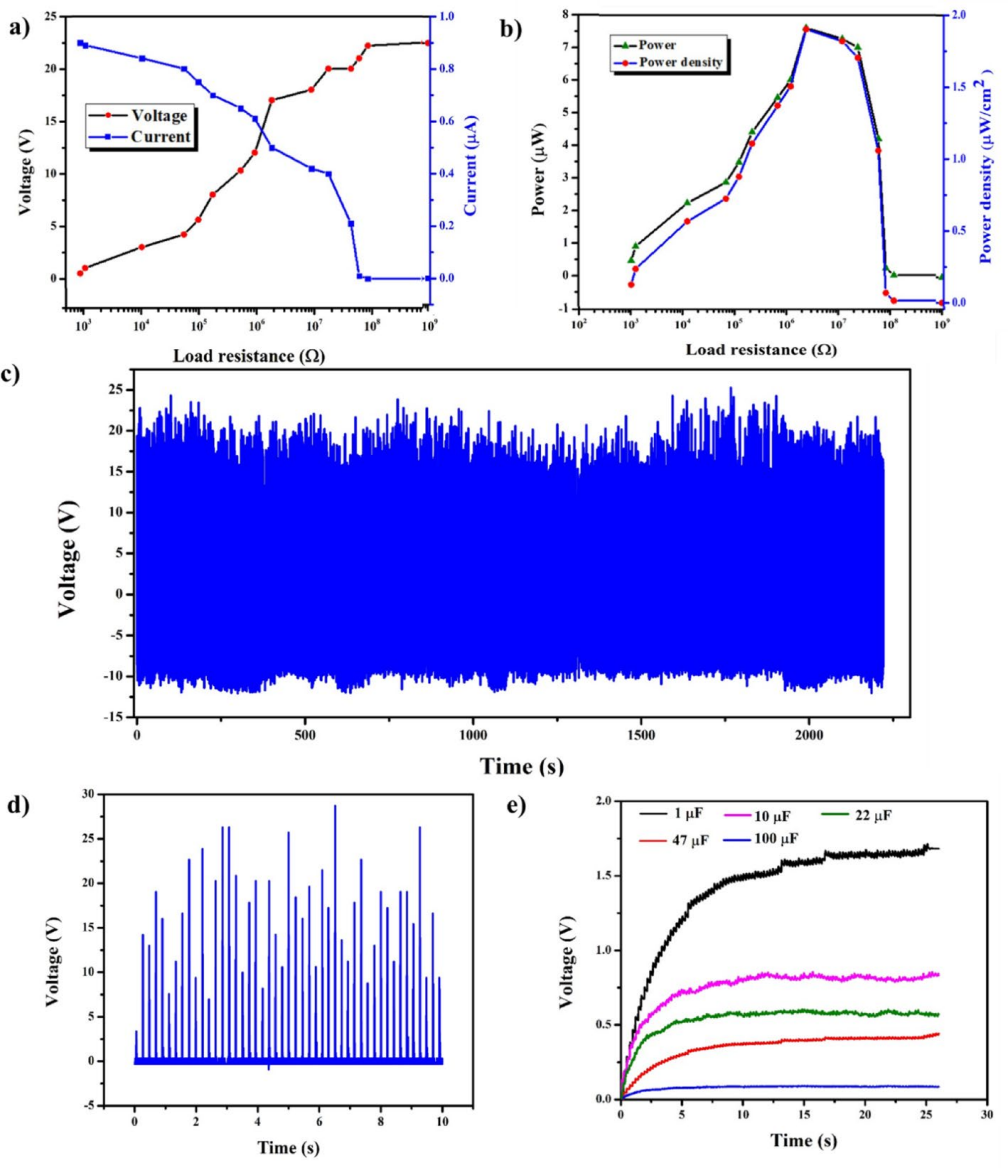
Detailed analysis of MS-TENG (**a**) Load analysis. (**b**) Power and power density. (**c**) Stability analysis. (**d**) Rectified output voltage. (**e**) Capacitor charging

To investigate the performance of MS-TENG in detail, a load and stability analysis is performed, as shown in Fig. 7. The load analysis is conducted to verify the stable and reliable power output at different load resistance values. Load matching maximizes the device’s efficiency. The range of resistors utilized here is 1 KΩ to 1 GΩ. The MS-TENG voltage increases with increase in resistance and vice-versa for the current, as shown in Fig. 7.a. The Instantaneous power is calculated using the formula,

1 $$ {\text{P }} = {\text{ V}}*{\text{I}} $$

where ‘V’ is voltage, and ‘I’ is current.

The maximum power obtained is 7.6 µW at 2 M Ω. Power density is calculated as,

2 $$ {\text{P}}_{{\text{d}}} = {\text{ Power}}/{\text{ area}} $$

The power density of the MS-TENG is 1.9 µW/cm², as shown in Fig. 7.b. Stability analysis is performed to determine whether the output remains stable over an extended period. From Fig. 7.c it is clear that the device output remains stable until 2250s confirming the suitability of MS-TENG for real-time applications. Figure 7.d shows the rectified output, as rectification is essential for utilising the output produced by the device. It enables compatibility with electronic devices and improves overall system efficiency. The pulsating AC is not suitable for real-time applications; therefore, it is rectified, and the negative parts are removed using a DF06G bridge rectifier IC, as shown in Fig. S9.

Capacitors are designed to carry out real-time applications described in Sect. 4. Various capacitors like 1 µF, 10 µF, 22 µF, 47 µF, and 100 µF are employed here to check the charge storage capacity of the device as depicted in Fig. 7.e. A 1 µF capacitor stores maximum voltage, which is nearly 1.6 V.

**Fig. 8:**
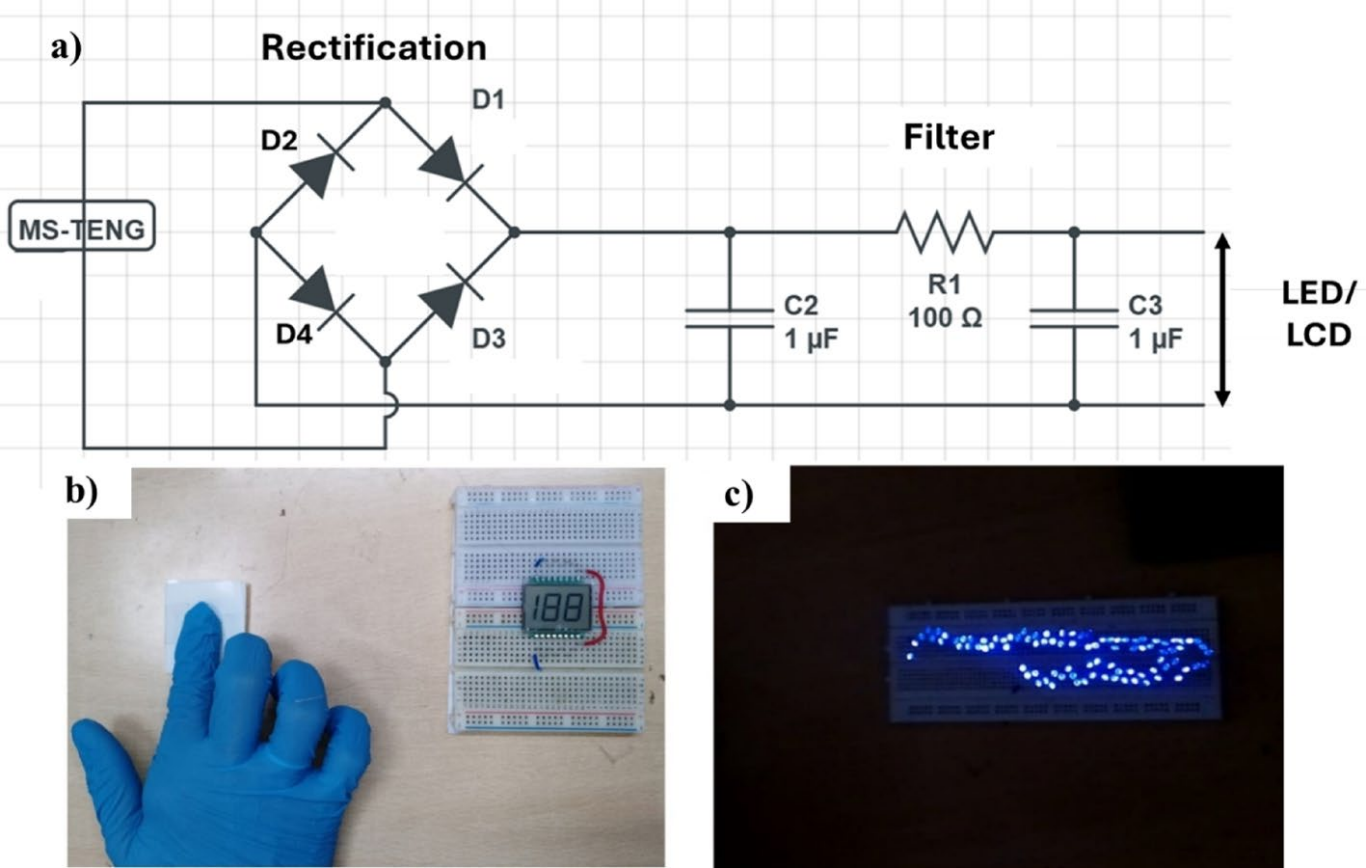
(**a**) Equivalent circuit for energy harvesting. Powering of (**b**) LCD (**c**) LEDs

The energy harvesting circuit in Fig. 8.a includes a DF06G bridge rectifier for AC-to-DC conversion, a capacitor bank (1 µF-100 µF) for charge storage and output stabilization, and passive RC filters to protect downstream electronics [29]. Powering of low-power electronic devices is shown in Fig. 8.(b-c), where LCD and LEDs are powered respectively. Please refer to the video V1.

## Applications of MS-TENG

MS-TENG can be used for different applications like bio mechanical energy harvesting during exercise (detailed analysis in SI Fig.S10 and please refer to the video V2) and power plant control simulation.

### Logic gates implementation

**Fig. 9:**
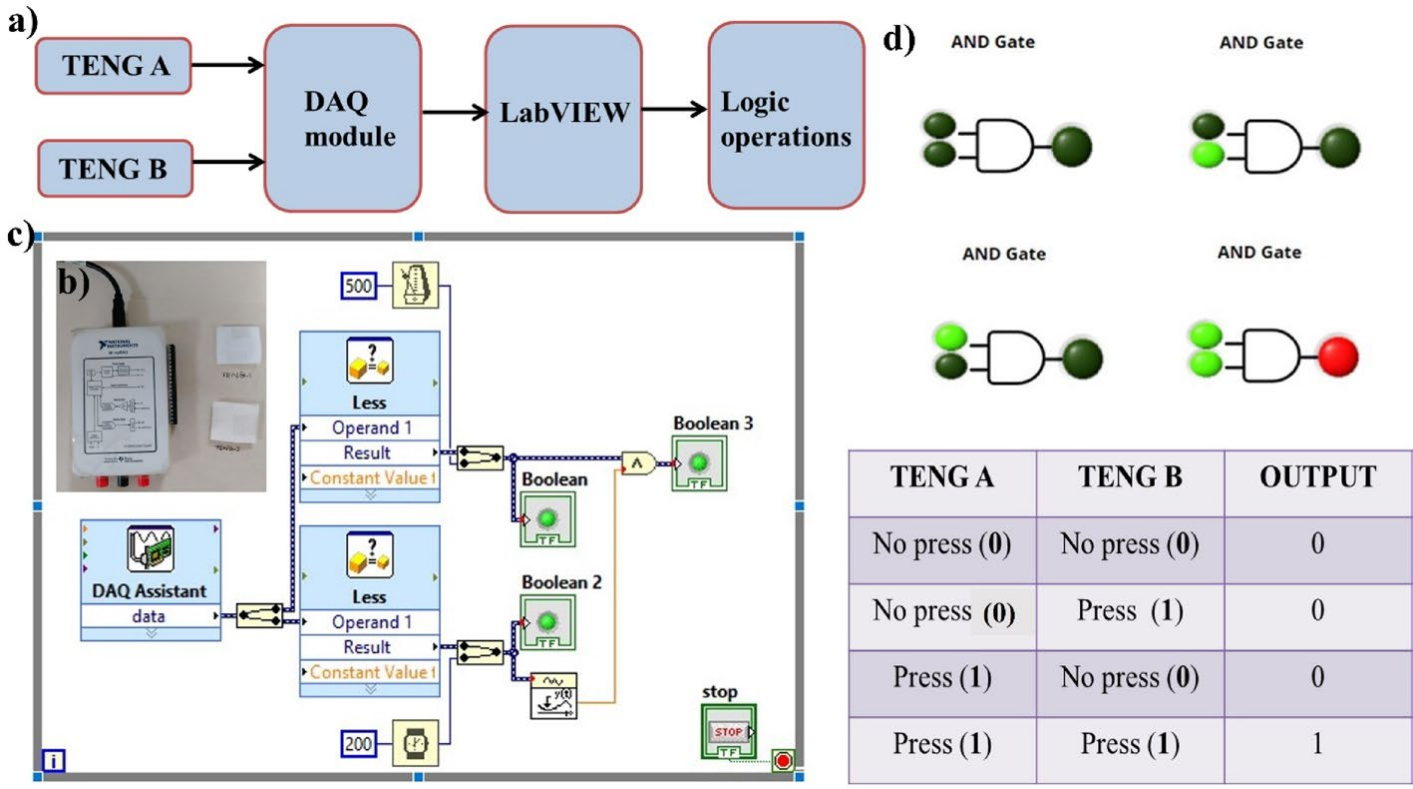
Logic operations implementation using MS-TENG as an input device. (**a**) Schematic representation of device set-up. (**b**) myDAQ module (**c**) Block diagram (**d**) Front panel of LabVIEW software

Logic gate operations were implemented before using a piezo phototronic principle where optical and piezoelectric properties were utilized [30]. In this work, an attempt has been made to implement logic operations using MS-TENG as an input device, integrating it with LabVIEW software. The schematic diagram is shown in Fig. 9.a, which consists of TENG devices, National Instrument’s NI myDAQ module, and LabVIEW software. MS-TENG A and B are connected to LabVIEW software via the NI myDAQ, which serves as an interface. The NI myDAQ is a compact, affordable electronic device, as shown in Fig. 9.b, with data acquisition capabilities that enable it to measure and analyze real-time signals. The hardware consists of analog I/O, digital I/O, audio, power supplies, and digital multimember ports. Two analog channels can be configured as either audio output or general-purpose voltage output. One can generate signals up to ± 10 V in general-purpose mode. The two channels in audio mode represent the left and right stereo outputs. Eight digital input/output pins are available, which can be programmed according to the application. The NI myDAQ can be interfaced with the graphical programming software Laboratory Virtual Instrument Engineering Workbench (LabVIEW). The internal architecture of a system can be represented in a block diagram, allowing the result to be displayed on the front panel [31].

The DAQ assistant is initialized by defining parameters such as sampling rate and voltage range. Building virtual instrument (VI) will then occur, and the DAQ module will be initialized as depicted in Fig. 9.c, which is the block diagram of the AND operation. To set a threshold, a comparison palette is used, and the input voltage is compared to follow the corresponding logic operations. Logic gate operations can be performed directly using the Boolean palette present in the block diagram of LabVIEW. TENG A and TENG B connected as input devices, with two conditions: PRESS i.e. in contact corresponding to ‘logic 1’ and ‘NO PRESS’ corresponding to ‘logic 0’. As shown in Fig. 9.d, the front panel displays the AND operation with a truth table. When both TENGs are in the ‘NO PRESS’ state, the output will be low. Similarly, any of the TENGs in ‘PRESS’ (LED blinking green) state output will be low. According to the AND operation, if both TENGs are in the ‘PRESS’ state, the output will be high (LED blinking red), as shown in the truth table. Please refer to the video V3.

Furthermore, logic gate implementation has been performed for NAND, OR, NOR, and XOR using TENG as the input device. Output has been obtained according to gate operations, depending upon the ‘PRESS’ and ‘NO PRESS’ states. Since logic gate implementation can be done, the same logic has been used to realize different logic operations (please refer to the video V4). A half-adder implementation is performed as an example. The block diagram consists of an XOR operation and an AND operation corresponding to the sum and carry, respectively. The digital logic is given as,

3 $$ {\text{Sum}} = {\text{ A}}`{\text{B}} + {\text{AB}}` = {\text{ A XOR B}} $$

4 $$ {\text{Carry}} = {\text{ A AND B}} $$

Where, A and B are inputs from TENG A and TENG B respectively.

In ‘NO PRESS ‘state, sum will be high, so XOR output LED will blink in red color. When both TENG A and TENG B in ‘PRESS’ state, carry will be high, thus AND output will blink in red color (video V4). Similarly, any logic operation can be realized using TENG as an input, using LabVIEW software.

LabVIEW is employed to simulate logic gate operations, with the MS-TENG serving as a binary input trigger, interpreting “PRESS” as logic 1 and “NO PRESS” as logic 0. The novelty is in the demonstration of the self-powered, gesture-responsive input capabilities of a triboelectric nanogenerator for digital logic simulations. This represents a significant advancement in the development of tactile human-machine interfaces. The LabVIEW integration validates a practical mapping between triboelectric signals and Boolean logic, which is pertinent for prototyping control systems, despite the fact that the logic gates are not physically implemented in hardware using TENG as a binary input device.

### Power plant control simulation

A programmable logic controller (PLC), also known as a programmable controller, is a ruggedized industrial computer designed to control manufacturing processes such as assembly lines, machines, robotic devices, or any activity that necessitates high reliability, ease of programming, and process fault diagnosis. Once it is integrated with SCADA, it can automate industrial processes with reasonable accuracy. Here, PLCs perform real-time control operations using signals from sensors and field devices. SCADA implements higher-level supervision, data acquisition, and information analysis. PLC and SCADA-based power plant control is being performed using ladder logic programming. It is a visual representation of operating machines and procedures. For instance, it can regulate the start and stop of a motor in a conveyor belt system by using sensor inputs. Ladder logic is a combination of various gate operations like AND, OR, NOT, and plays a vital role in industrial automation. The execution of the program is based on real-time input from the industry, and basic programming consists of a ladder-like structure with horizontal lines- ‘rungs’ and vertical lines – ‘rails’ [32]. Series or parallel connections are given to the inputs, and the output is connected in parallel. The operation of ladder logic diagram is both top-down and left-to-right. The associated output coil becomes activated when all a rung’s conditions are satisfied. Then, it initiates the system in the real world, such as turning a motor on or off. To guarantee operating system automation, the PLC and control system repeatedly scan and repeat this operation [33].

**Fig. 10:**
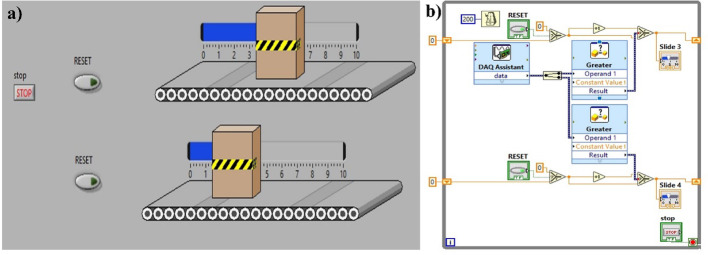
Power plant control simulation- (**a**) Conveyor belt control front panel (**b**) Block diagram

**Fig. 11:**
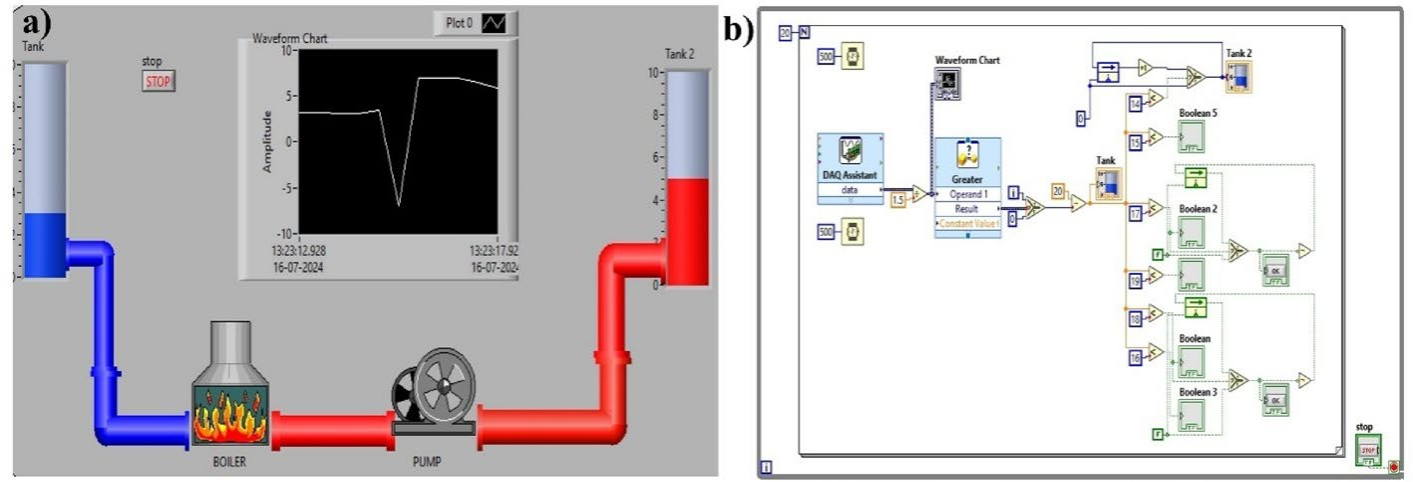
Power plant control simulation- Coolant control (**a**) front panel and (**b**) block diagram

A similar approach is employed here, utilizing the ladder programming concept. A combination of different logic gates is implemented to execute power plant control operations with TENG as the input device. LabVIEW software is used to control parameters such as conveyor belt motion and coolant control in a power plant, as shown in Fig. 10a-b, respectively. MS-TENG is acting as an input device (trigger device) in both cases. When the device is hand-tapped, the object placed over the conveyor starts moving, 2 cm (Fig. 10.a) in each tapping depicting human machine interface. After reaching the endpoint, the process can be reset, and the object can be brought back to its initial position. The corresponding block diagram shown in Fig. 10.b, consists of a select switch, a shift register, and a comparator. When the incoming voltage value from TENG exceeds the threshold, a specific condition is selected, which is the movement of an object. Similarly, the coolant control in the power plant is also illustrated in Fig. 11.a.

Initially, the liquid was in a cold state, indicated by its blue color, and it turned red as it was heated using a boiler in the plant. When water is poured into the glass and placed over MS-TENG, the simulated tank A becomes full, and thereby the valve opens. Thus, the boiler begins to work, converting cool water into hot water, followed by a centrifugal pump. Further, the hot water will be stored in tank B, which is triggered by the MS-TENG. The corresponding block diagram is shown in Fig. 11.b, which includes a comparator and signal conditioner. Please refer to the video V5. The LabVIEW simulations were created as a proof-of-concept to illustrate the potential of MS-TENG-generated signals to function as triggers for ladder logic operations, which are the foundation of PLC and SCADA systems. Although the mapping is intentionally simplified, it effectively demonstrates event-driven control by converting mechanical energy into discrete digital states that initiate industrial sequences. Nevertheless, the binary interpretation of “PRESS” as logic 1 and “NO PRESS” as logic 0 is a recognised limitation of this approach. This interpretation oversimplifies the complexity of real-world control dynamics, which typically necessitate continuous feedback and multi-sensor integration. In this way, MS-TENG is a novel member in power plant control operations and other potential industrial applications, offering a cost-effective and environmentally friendly solution.

## Conclusion

In brief, a MIL-53 incorporated sponge-based triboelectric nanogenerator is proposed. The crystalline structure of MIL-53 was examined with XRD. FTIR spectroscopy was used to determine the functional groups present in it. The surface morphology, including particle size and shape, was studied using FE-SEM. The surface charge of MIL-53 particles was evaluated using zeta potential analysis. Ecoflex polymer is used to prepare sugar sponge, within which the MOF particles are impregnated. Pristine sponge and MIL-53 sponge are utilized to form PS-TENG and MS-TENG in contact separation mode. The highest electrical response was obtained for the MS-TENG with a 4 wt% filling of MIL-53, and the maximum voltage, current, and transferred charge were 44.2 V, 0.9 µA, and 6 nC, respectively, which is 147% compared to a pristine sponge TENG. The device remains stable for up to 2250 s with the same output performance, thus the MS-TENG is capable of real-time applications. The device is used to power LEDs and LCDs, and logic gate implementation has been performed with TENG as the input device. Finally, MS-TENG was connected to LabVIEW software to perform power plant control simulation, implementing logic gate operations. Two conditions are defined based on the pressing, logic 0 to ‘NO PRESS’ and logic 1 to ‘PRESS’. All gate operations are realized using this setup, and a half adder is also implemented. Since ladder logic is the basis of PLC-SCADA-controlled power plant operations, MS-TENG can be used as a trigger device in simulating this. In this way, operations like conveyor belt control and coolant control are also implemented here.

## Supplementary Information

Below is the link to the electronic supplementary material.

Supplementary Material 1

## Data Availability

Data is provided within the manuscript and supplementary information files. The raw files will be available upon demand/request. This manuscript does not use data generated from other studies. This is an original research.
