# Supplementary material for: Metal-organic framework impregnated sponge-based TENG as a binary input device for logic gate simulation and power plant control
Source: Discov Nano. 2025 Oct 30;20(1):196. doi: 10.1186/s11671-025-04369-6 (PMC12575916; doi:10.1186/s11671-025-04369-6)
Supplement: Supplementary file 1 — Supplementary Material 1 [file 11671_2025_4369_MOESM1_ESM.docx]

**Supporting information**

**Metal-organic Framework Impregnated Sponge based TENG to Implement Logic Gates Operations and Power Plant Control Simulation**

Nitha P K ^a^, Gaurav Khandelwal ^b^, Arunkumar Chandrasekhar ^a*^

^a, a*^ Nanosensors and Nanoenergy Lab, Biomedical instrumentation Lab,

Department of Sensors and Biomedical Technology, School of Electronics Engineering,

Vellore Institute of Technology, Vellore, Tamilnadu, India

^b^ Engineering and Technology Institute (ENTEG), University of Groningen, Groningen, Netherlands

Corresponding author's Email:

^*^[arunkumar.c@vit.ac.in](mailto:arunkumar.c@vit.ac.in)

Dr. Arunkumar Chandrasekhar

Nanosensors and Nanoenergy Lab, Biomedical instrumentation lab,

Department of Sensors and Biomedical Technology, School of Electronics Engineering,

Vellore Institute of Technology, Vellore, Tamilnadu, India

ORCID ID-https://orcid.org/0000-0002-4561-0975

**

**

**Fig.S1.** SEM image of MIL-53 synthesized in 150ºC for 15 hours


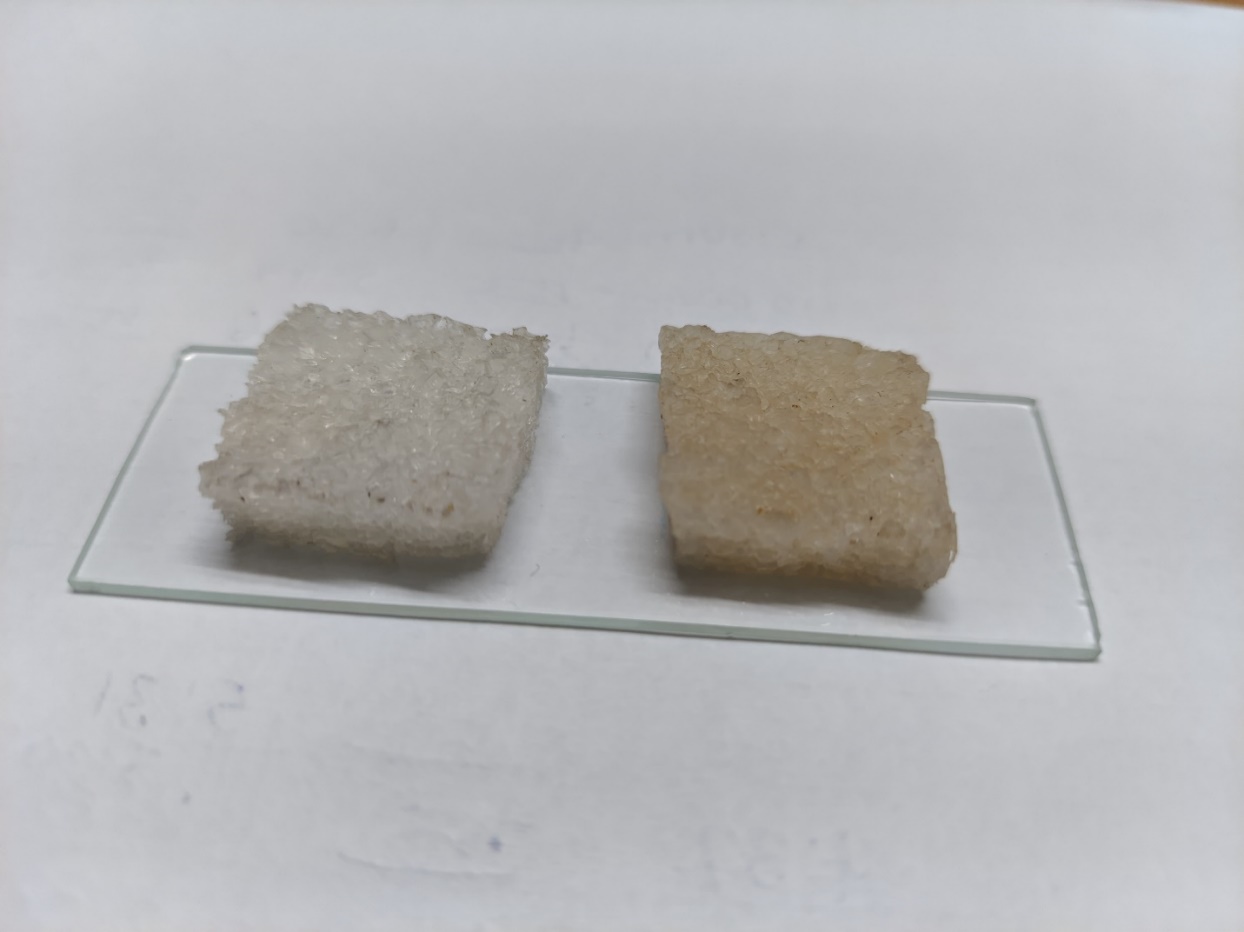


**Fig.S2.** Real image of pristine and MIL-53 sponge

**
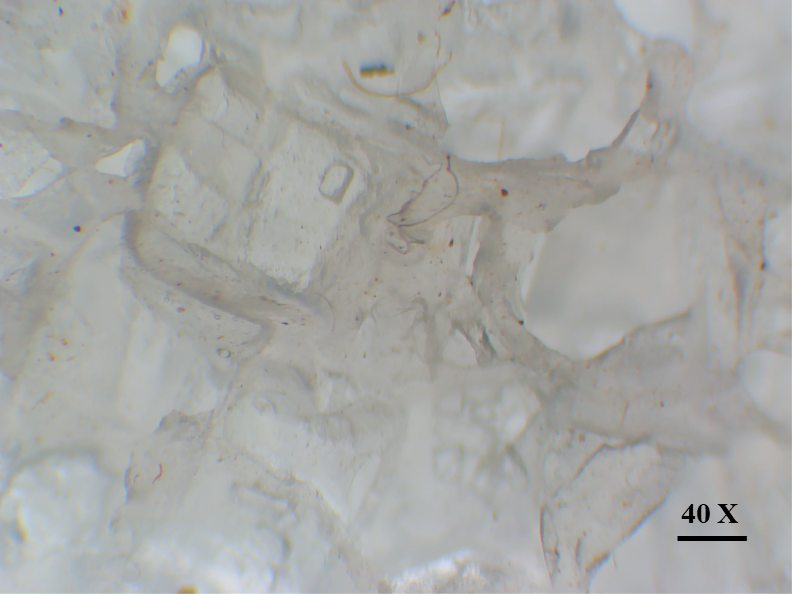
**

**Fig.S3.** Microscopic image of pristine sponge


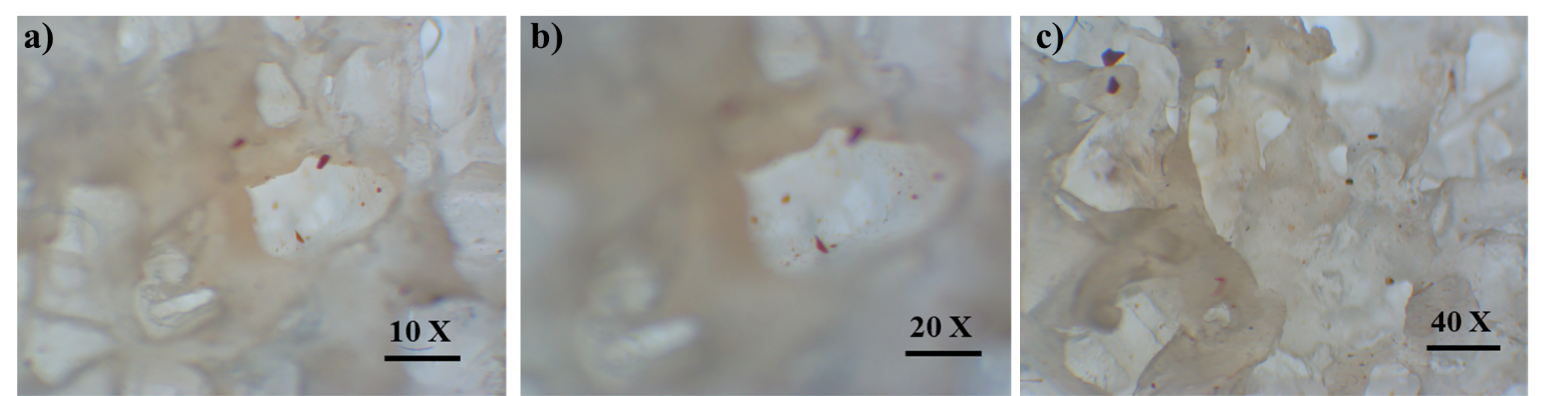


**Fig.S4.** Microscopic image of MIL-53 sponge- different magnification-a)10x b)20x c)40x





**Fig S5**. SEM image of sponge after MIL-53 impregnation


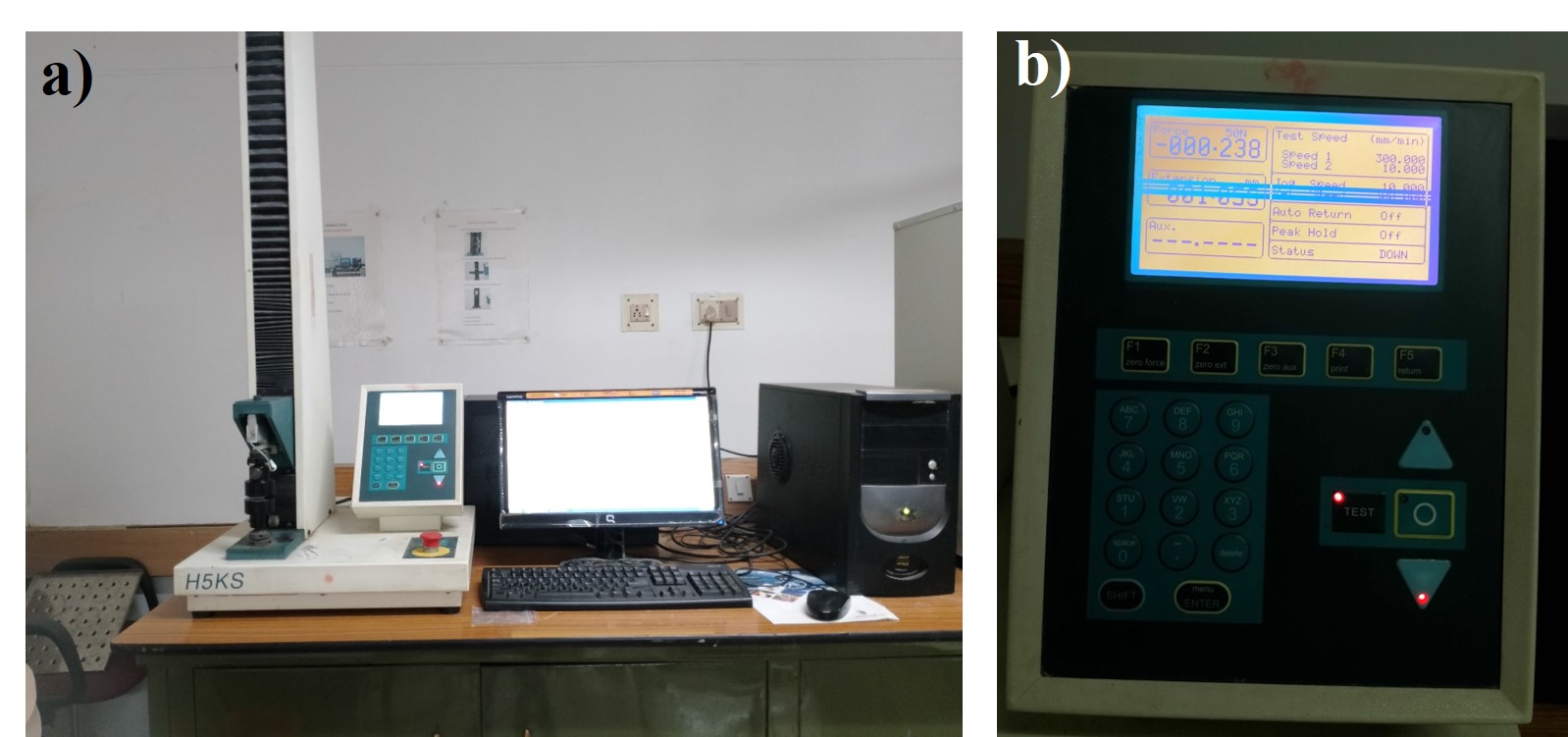


**Fig. S6.** Compressibility check arrangement. a) Universal testing machine. b) Parameter set-up


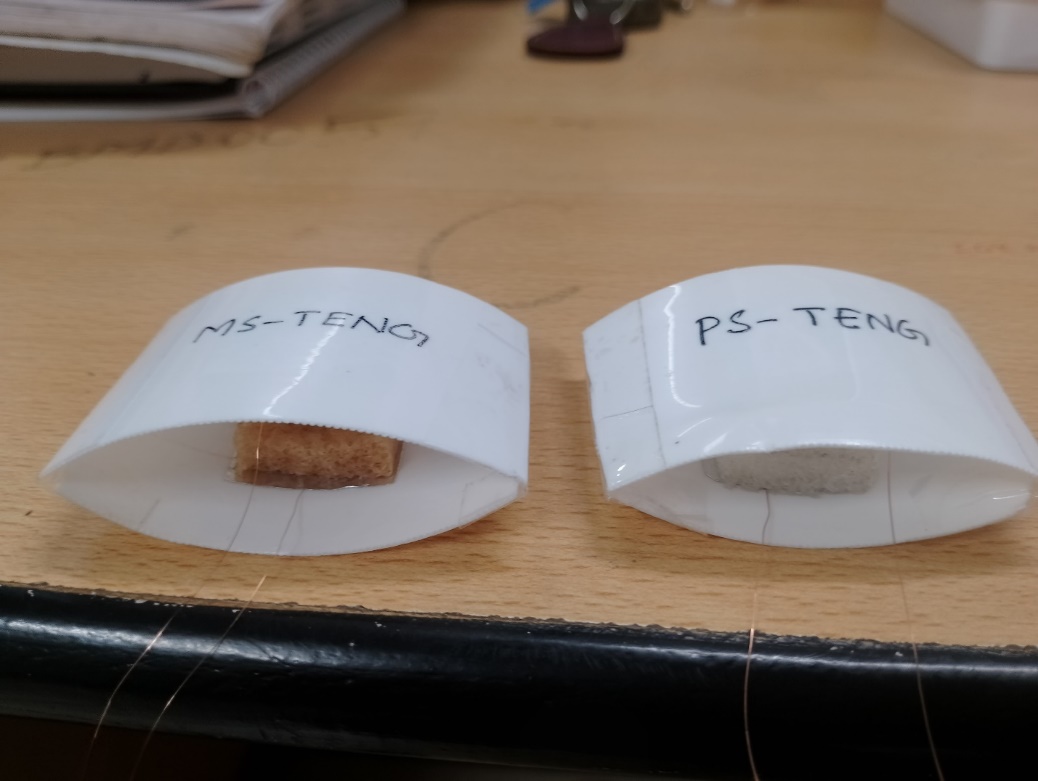


**Fig. S7**. Digital image of MS-TENG and PS-TENG


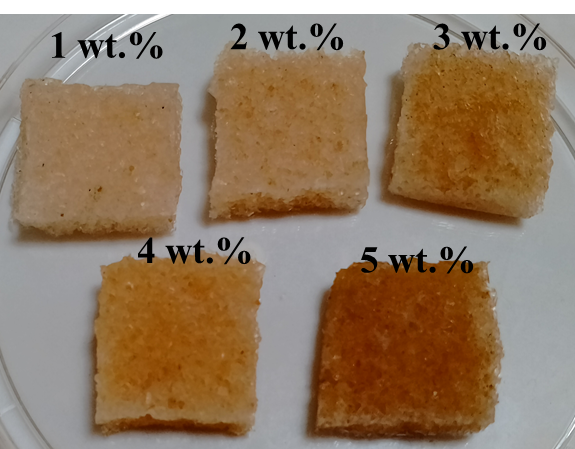


**Fig S8**. MIL-53 sponges with different wt. % filling of MOF


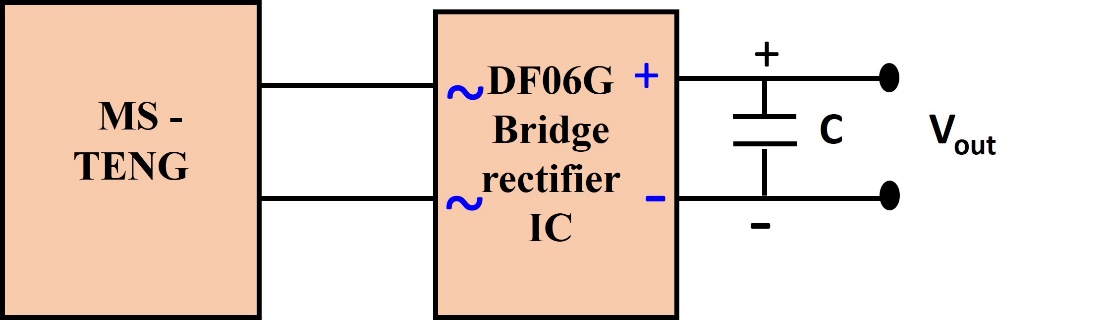


**Fig S9**. Equivalent circuit diagram

- 1. **Exercise monitoring**

MS-TENG can be employed for biomechanical energy harvesting by body movements. Thus it is a suitable to perform exercise monitoring [1]. Here MS-TENG is utilized for monitoring biceps curls, triceps extension and front arm raises by analyzing the maximum frequency value obtained by fast Fourier transform signal processing [2]. It can be used to count the number of peaks during different exercises posture is correct, corresponding frequency peaks will be obtained each time. The device is placed as shown in Fig. S.10 and in the biceps an output voltage of 1.5 V obtained. Similarly, energy harvested in triceps extension and front arm raises and obtained a voltage of 3 V and 1.25 V respectively. For biceps curls the peak is corresponding to 0.96 Hz, and in triceps extension one major peak in 1.62 Hz is present as depicted in Fig 8. c and f respectively. The frequency peak of front arm raises is different from other two peaks and present in 0.2 Hz. In this way the proposed device can be utilized for exercise monitoring in a simple and cost-effective manner. Please refer to video V2.


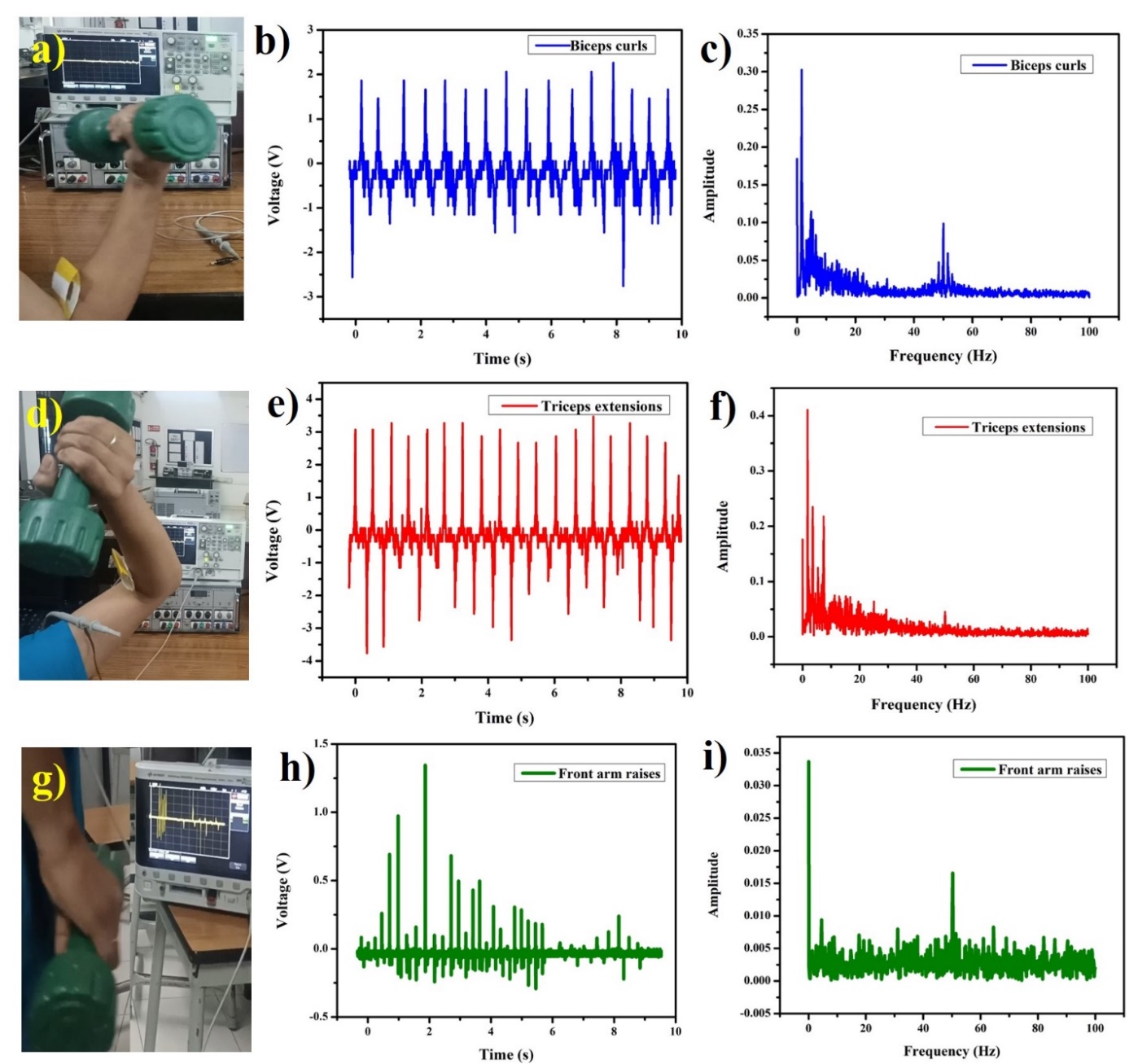
**Fig.S10. Exercise monitoring using MS-TENG.** a-c) Biceps curls. d-f) Triceps extensions. g-i) Front arm raises.

**Table S1.** Optimization of temperature and time for MIL-53 synthesis

| **Sl No** | **Precursors** | **Temperature** | **Reaction time** | **Product formation** |
| --- | --- | --- | --- | --- |
| **1** | 2.45 mmol FeCl_3_.6H_2_0 + 1.24 mmol Terephthalic acid | 120ºC | 24 Hour | No, mixture of precursors. |
| **2** | 2.5 mmol FeCl_3_.6H_2_0 + 2.5 mmol Terephthalic acid | 150 ºC | 15 Hour | No, mixture of precursors. |
| **3** | 2.5 mmol FeCl_3_.6H_2_0 + 2.5 mmol Terephthalic acid | 150 ºC | 24 Hour | **Yes, crystalline and pure product formed.** |

References

[1] G. Khandelwal, N. P. Maria Joseph Raj, and S. J. Kim, “ZIF-62: a mixed linker metal-organic framework for triboelectric nanogenerators,” *J. Mater. Chem. A*, vol. 8, no. 34, pp. 17817–17825, 2020, doi: 10.1039/d0ta05067a.

[2] A. Chandrasekhar *et al.*, “Smart maracas: An innovative triboelectric nanogenerator for earthquake detection and energy harvesting,” *Nano Energy*, vol. 123, p. 109379, May 2024, doi: 10.1016/j.nanoen.2024.109379.
